# Case of Disordered Action of the Heart, with Dropsical Effusion, Relieved by Blood-Letting

**Published:** 1817-08

**Authors:** Edward Bourne


### Medico-Chirurgical

### Journal and Review.

VOL. IV.] AUGUST, 1817. [NO. 20.

### PART I.

### ORIGINAL COMMUNICATIONS.

——— quid utile ———

### *Case of disordered Action of the Heart, with Dropsical Effusion, relieved by Blood-letting.*

By EDWARD BOURNE, M. D.*

OCTOBER 20th, 1815. I was requested to visit Mrs. F. ætat. 22, residing about ten miles from this city. She complained of much difficulty of breathing, and palpitations of the heart, with an almost constant sensation of fluttering within the chest and at the epigastrium. Had frequent sighings; little or no cough or pain, excepting slight transient shoots through the breast, occasionally ex-

* Dr. Bourne, in communicating this very instructive case, writes thus to the Editors:

" GENTLEMEN,

" Having seen in the *Medico-Chirurgical Journal* for last month, an account of a case published, in the second part of the first volume of the *Transactions of the Medical Society of London*, by Dr. Clutterbuck, entitled, '*Disordered Action of the Heart, &c. effectually relieved by Blood-letting and the horizontal Posture,*' it brought to my recollection one of a similar nature, as to its general character, which fell under my care in the autumn of 1815. You will, herewith, receive tending to the left arm. The pulse extremely feeble, and, at the same time, the most irregular and intermittent I ever felt: insomuch that a distinct beat could be reckoned only from every two to four seconds; intermediately, sometimes fluttering and undulatory vibrations; sometimes no palpable motion at all. The countenance was anxious; face bloated and pale, excepting a circumscribed flush on each cheek. Tongue whitish at the back part; the edges and fore part sore and abraded. Thirst. Surface of the body very pale; skin cool; legs very œdematous; urine scanty; bowels open by medicine. She could lie only on her back with the shoulders raised; and got but little sleep: was now sitting in a chair. I learned that she had been delivered of her first child six weeks past, had a good time, and went on well till a week ago, when she was seized with rigors succeeded by heat, accompanied with sharp pain about the region of the heart, and some pains likewise in the bowels. The febrile symptoms subsided in three or four days, and the pains left her.

On the 17th, her pulse was found to be irregular and intermittent: the dyspnœa, palpitations, fluttering, and œdema, also then first attracted attention, and have since kept increasing. She has naturally a florid healthy look; but was formerly subject to temporary oppressed breathing upon extraordinary exertion.

As I had never before witnessed so feebly irregular, and altogether apparently alarming state of the circulation, I was somewhat puzzled how to act in the first place; yet

---

such a statement of it, as was noted at the time of its occurrence, without any expectation then of its ever meeting the public eye. Notwithstanding, if, upon perusal, you consider it worthy a place in your Journal, you are at liberty to give it publicity.

" I am your's, &c.

" *Coventry, July* 1st, 1817. EDWARD BOURNE."

Some cases, farther illustrative of the bold and successful practice of Drs. Clutterbuck and Bourne, will probably be given, in the course of next year, by one of our Editors, who is arranging materials, of several years' accumulation, for a paper " *On the Efficacy of Venæsection and Abstinence in several morbid Affections, hitherto erroneously regarded as Diseases of Debility.*" See Dr. Johnson's Communication in the first number of the *Medico-Chirurgical Journal;* and the *Cases of Diabetes,* by Dr. Watt, of Glasgow; by the perusal of whose interesting work our attention was first aroused to this subject; and who, we are not without hope, will, through the medium of our Journal, favour the public with farther evidence of his success in this almost untrodden path of practice. We anxiously exhort the bigots of the Brunonian school to rub the dust from their blind eyes, and ponder over these things! EDITORS.

the symptoms seemed to demand prompt assistance. I considered the case to be *hydrops pericardii,* and that probably also the heart had itself sustained some disorder in its organization, from previous inflammatory attack: that viscus appeared to be constantly, but ineffectually, labouring to discharge itself of its accumulated load. Hence, the palpitation, sense of fluttering, &c. There was every external mark of *defectus sanguinis quoad molem;* nevertheless, probable evidence of internal *plethora ad vires,* in the pulmonary circulation especially. A question then occurred:—Might not the abstraction of a portion of blood from the venous system assist the heart, and relieve dyspnœa and other distressing symptoms? But, the remedy was new; and, independently of the above reasoning, every feature of the disease seemed to forbid the use of the lancet. My visit was late in the evening; and having an opportunity of sleeping at a friend's house in the village, I was induced to make trial, until morning, of some medicines gently stimulating to the stomach, with a view likewise to their determination to the skin and kidnies. The following were therefore prescribed:

℞. Pulveris opii gr. j; scillæ gr. x; hydrargyri submuriatis gr. vi; syrupi q. s. s. fiant pilulæ viij. Capiat duas horis singulis tertiis, cum haustu sequente.

℞. Potassæ acetatis ʒß; spiritus etheris sulphurici ʒj; misturæ camphoræ ℥iß, fiat haustus. Imponatur sterno emplastrum lyttæ satis amplum.

On the morning of the 21st, we found she had had a little sleep, with some perspiration. No stool. General symptoms the same as last night. I now requested the attending surgeon to open a vein in the arm. The circulation was so extremely languid, that, notwithstanding the orifice was sufficiently large, the blood issued only by drops; till, by friction, and immersing the arm in warm water, we obtained a stream. I kept my finger at the opposite wrist during the flow. From six to eight ounces were abstracted. During the operation, our patient expressed relief to her breathing, to the palpitations, &c.; and the pulse gained a little more freedom and regularity. Omittantur medicamenta.

℞. Submuriatis hydrargyri gr. vi; pulveris antimonialis, sacchari āā ℈j; misce et divide in chartas vj. Sumat. unam 4tis vel 5tis horis, cum coch. tribus amplis solutionis magnesiæ sulphatis ʒvj ex decocti hordei ℥viij.

℞. Tincturæ camphoræ comp. spiritus ether. sulphurici

āā ʒj; misturæ camphoræ ℥vj; liquoris ammoniæ acetatis ℥ß; M. sit haustus, nocte, hora somni capiendus.

The bleeding was directed to be repeated in the evening, unless there were an amendment of the symptoms. She was enjoined to remain in bed, and to be kept quiet; and to have a light, principally farinaceous, diet.

22d, hora 7ma P. M. From three to four ounces of blood were with difficulty taken away last night. She had some composed sleep, and feels occasionally rather better; but the pulse and pectoral symptoms are now much the same. She has perspired a little, and had several liquid stools. The blood drawn exhibits no signs of inflammation, nor any thing unusual, in the proportion or quality of the crassamentum and serum. Blister has risen. Venæsection was now repeated to ℥viij, with relief as before. Continuentur pulv. et solutio ad unam vel duas alteras vices. Hora somni habeat haustum insequentem, vice prioris.

℞. Tinct. camphoræ comp. ʒij; spirit. etheris nitrici ʒj; misturæ camphoræ ʒx, fiat haustus.

23d, hora 8va. A. M. Has had more sleep in the night, with profuse perspiration; pulse still very irregular, but beats with more freedom; and she feels, on the whole, less of the palpitations and other distressing symptoms within the chest: thinks the bleedings have relieved her, and she has borne them very well. Belly continues open: urine in rather larger quantity, high coloured, and depositing a pinky sediment. Repetatur venæsectio ad ℥viij.

℞. Pulveris antimonialis ℈j; potassæ nitratis ʒj; misce et divide in sex chartas. Capiat unam sextis horis cum haustu è misturæ camphoræ ℥iß; spiritus etheris nitrici ʒj. Hora somni repetatur haustus ut hesterna nocte. Solutio magnesiæ sulphatis, si opus sit.

24th. Breathing and palpitations better; pulse much as yesterday. One free natural stool; urine diminished again. The circumscribed flush of the cheeks nearly gone. Tongue more natural; still thirsty. Quamprimum repetatur solutio purgans. Continuentur 5tis vel 6tis horis, pulveres heri scripti, cum haust. sequente, vice prioris.

℞. Infusi digitalis ℥ß; tincturæ scillæ gtt. xx; spiritus etheris nitrici ʒj; aquæ cinnamomi ℥j; misce sit haustus. Omittatur haustus anodynus.

26th. Pectoral symptoms much relieved; pulse now but slightly intermittent or irregular, of moderate strength and frequency. Copious secretion of urine, without sediment. Tongue better and thirst less; bowels open. Some return of appetite. Troublesome cough came on yesterday, for which she had a mixture of oxymel scillæ, tinctura camphoræ composita et aqua. Repetatur emplast. lyttæ thoraci. Continuentur medicamina omnia ut antea.

29th. Very little remains now of the dyspnœa, palpitations, or fluttering. Pulse nearly regular, and of good strength; but there is still some variableness and intermission. Free secretion of urine continues; no sickness; tongue nearly natural; appetite good; sleeps well. She can now lie down much better, and easily on either side. Five stools to-day. The powders had been changed by the omission of the nitrate of potash, and substitution of a small portion of rhubarb. Cough better. Intermittantur pulveres. Continuentur haust. infusi digitalis, &c.

30th. A good night, and all circumstances as well, or rather improved. Continuentur haustus ex infus. digitalis, &c. Repetantur pulveres pro re natâ, alvo nimirum pigrâ.

From this period my visits were discontinued, but I was informed by the family surgeon, that by a continuance of the remedies she proceeded in her recovery. Some weeks afterwards I called, and found her apparently well, going, as formerly, about her domestic concerns; in fact, complaining of nothing. However, upon feeling her pulse, there was some irregularity and intermission. I endeavoured to impress upon her the necessity of doing something more, and that she was by no means so well as she fancied herself. My representations were not then attended to. In the latter part of the ensuing spring, I accidentally heard of her having become ill again, and gone to place herself under the care of an empiric. From thence she had returned but a day or two, when a messenger came one afternoon to desire I would again visit her. The account given was, that she was then in a similar situation to what I had first seen her in. I could not conveniently go that evening, but promised to see her early the next morning. Accordingly, I set off about six o'clock, and had not proceeded a mile, when a servant met me to say Mrs. F. had died in the night.

In this case, I think we may say with Dr. Clutterbuck; it was one "which seemed of all others, from general appearances, to be most unfit for bleeding, but which not only bore that evacuation with impunity, but was relieved by it."

To what extent the bleedings contributed to the recovery of the patient, may, perhaps, admit of doubt; nevertheless, they alleviated some of the most distressing symptoms, and evidently did no harm. The disease, it is true, yielded but little, till the digitalis, &c. effected a copious secretion of urine. The improvement was then rapid. But, might not the repeated abstraction of blood prepare the system for a better and more decided action of the diuretics? This is not the only instance wherein I have had reason to believe that the action of diuretics hath been more prompt and efficient, consequent to the employment of blood letting, either general or local: and I am of opinion the practice might, with due precaution, be beneficially extended to many cases of every species of dropsy.

